# Quality improvement in emergency units in low resource settings: a qualitative assessment for understanding challenges and delivering solutions

**DOI:** 10.1136/bmjoq-2026-004186

**Published:** 2026-08-05

**Authors:** Indrakantha Welgama, Daniel Lange, Gerard O’Reilly, Aurore Nishimwe, Agnieszka Ignatowicz, Justine Davies

**Affiliations:** 1 Department of Applied Health Sciences, College of Medicine and Health, University of Birmingham, Birmingham, UK; 2 School of Public Health and Preventive Medicine, Monash University, Melbourne, Victoria, Australia; 3 King’s Centre for Global Health and Health Partnerships, King’s College London, London, UK

**Keywords:** Global Health, Emergency department, Quality improvement, Qualitative research

## Abstract

**Objective:**

This study aimed to identify the barriers and facilitators for quality improvement (QI) in emergency units (EUs) in low- or middle-income countries (LMICs) and to understand the prioritisation of care quality domains to inform the development of EU-specific guidance for QI across all World Health Organisation (WHO quality domains for LMICs.

**Methods:**

We conducted in-depth interviews (IDIs) with specialist emergency physicians from LMICs and convened a workshop with global technical experts in emergency care. A structured topic guide was used for the IDIs. Interviews were conducted virtually, transcribed and thematically analysed using the WHO quality domains and the WHO Health Systems Framework. Data collection and analysis continued until saturation was reached. The 2-day workshop included presentations, discussions and interactive ranking exercises to assess measurements for QI and prioritise WHO quality domains for guidance development. Findings from both components were synthesised to generate key insights.

**Findings:**

Eleven IDIs were conducted and 26 experts participated at the workshop. QI efforts in LMIC EUs primarily focused on quality domains of effectiveness, timeliness, safety and occasionally patient-centredness. Key barriers to QI included high patient turnover, a lack of skilled staff, limited funds and resources and prioritising patient care quantity over quality. Facilitators included the availability of QI guidance, training, initiating small-scale projects, enhancing documentation and communication and supportive hospital management. Safety and effectiveness were considered the most feasible domains for QI, although all domains were considered important. Variability in how participants defined these domains highlighted the need for clear guidance on definitions, their relative importance and appropriate measures for each.

**Conclusions:**

EUs are complex, high-pressure environments managing time-critical health conditions and require development of specific tools and guidance for QI. Addressing the barriers and leveraging facilitators identified in this study can inform the development of effective QI strategies for LMIC EUs.

**WHAT IS ALREADY KNOWN ON THIS TOPIC**

- Available evidence suggests that poor quality of care in health facilities is a substantial driver of adverse health outcomes, with a growing concern about the prevalence of avoidable mortality and morbidity associated with emergency health conditions in low- or middle-income countries (LMICs).
- The World Health Assembly resolution 76.2 (2023) called for strengthening of emergency, critical and operative care (ECO care) services, including enhancing care quality especially in emergency units, which are the primary point of contact in health facilities for patients with time-critical conditions.

**WHAT THIS STUDY ADDS**

- This study reflects on the types of quality improvement activities carried out within emergency units in LMICs and identifies key barriers and facilitators influencing quality improvement, underscoring the need for tailored guidance and tools.
- It proposes potential for assessing and addressing quality of care across all seven WHO quality domains within the context of emergency units, especially in LMICs.

**HOW THIS MIGHT AFFECT RESEARCH, PRACTICE OR POLICY**

- Our study findings provide a foundation for developing emergency unit-specific guidance and tools for quality assessment and improvement, particularly for resource-constrained settings, contributing to the broader goal of achieving the objectives outlined in World Health Assembly resolution 76.2 (2023).

## Introduction

 Low- or middle-income countries (LMICs) encounter a large burden of emergency conditions,^1 2^ contributed to by a high incidence of injuries, infectious diseases and complications of non-communicable diseases.^1^ Disability affected life years attributed to emergency conditions are greatest in low-income countries followed by middle-income countries.^3^ Prior studies have identified that approximately 40% of deaths from injuries and other time-critical conditions are avoidable; with 60% of these avoidable deaths occurring due to issues in receiving good quality care in health facilities.^4 5^ These findings for emergency conditions align with the findings of Lancet Global Health Commission on High Quality Health Systems, which suggests that poor quality of care in facilities is a substantial driver of adverse health outcomes.^6^ In LMICs, similar to high-income countries (HICs), patients with emergency conditions often present to emergency units (EUs)^7^; however, mortality in LMIC EUs far exceeds that in HICs.^6 8–10^

Quality healthcare is experienced across multiple domains, as defined by the Institute of Medicine^11^ and updated by the WHO.^12^ These quality domains include effectiveness, safety, people-centredness, equity, efficiency, timeliness and integrated care, as follows.^12^

- Effective: ‘providing evidence-based healthcare services to those who need them’.
- Safe: ‘avoiding harm to people for whom the care is intended’.
- People centred: ‘providing care that responds to individual preferences, needs and values’.
- Equitable: ‘providing care that does not vary in quality on account of gender, ethnicity, geographic location, and socio-economic status’.
- Efficient: ‘maximizing the benefit of available resources and avoiding waste’.
- Timely: ‘reducing waiting times and sometimes harmful delays’.
- Integrated care: ‘providing care that makes available the full range of health services throughout the life course’.

Addressing all seven domains of quality healthcare is essential for successful universal health coverage,^13^ and guidance to assist providers with improving quality of care is developed by WHO.^14^ However, while quality improvement (QI) initiatives have been widely promoted, their implementation in LMICs is challenging.^15 16^ There also remains a gap on how QI in all quality domains can be operationalised in EUs, particularly in LMICs.

Prior studies in LMICs highlight multiple deficiencies in health facility structures and processes which could lead to poor quality outcomes. These issues include the lack of skilled healthcare providers, lack of protocols, under-developed information technology and data collection, poor access to essential medicines and equipment and financial constraints and governance issues; as well as affecting quality per se, these issues also impede QI efforts.^17–20^ Although the challenges in providing high quality healthcare and delivering QI in LMICs are well described, there is limited knowledge of QI implementation in EUs. EUs represent complex, high-pressure environments characterised by high patient volumes, diverse case mixes and the need for rapid decision-making and intervention within short clinical encounters.^21^ These contextual factors may amplify existing system-level challenges to effective QI implementation.

World Health Assembly resolution 76.2 of 2023 on ‘Integrated emergency, critical and operative (ECO) care for universal health coverage and protection from health emergencies’ was ratified in response to challenges affecting patients in need of emergency care.^22^ Subsequently, the WHO has been requested a provide a global strategy and action plan to assist in the implementation of this resolution.^23 24^ QI initiatives within the EUs will be central in achieving resolution 76.2, since EUs receive most emergency, critical and operative (ECO) conditions presenting to health facilities. Very few studies have assessed the QI interventions specifically in the EUs, and most of them tend to prioritise timeliness, safety and effectiveness, often through the use of standardised protocols and initiatives to improve system efficiency.^17 25–29^ However, other WHO quality domains, such as patient-centredness, equity and efficiency, are less consistently addressed, likely due to the operational challenges of delivering rapid, high-volume, time-critical care in resource-constrained settings. As a result, achieving a holistic approach to quality of care remains challenging. There is limited understanding of the specific barriers to implementing QI across all WHO quality domains in EUs in LMICs, as well as how these challenges can be addressed.

To inform the development of ECO care-related guidance for QI delivery in LMIC EUs, this study aimed to identify barriers and facilitators to implementing QI, and the prioritisation of care quality domains for QI guidance development.

## Methods

This study was conducted in two phases. First, we conducted a qualitative study consisting of in-depth interviews (IDIs) to explore barriers and facilitators affecting QI delivery in EUs in LMICs. Second, we hosted an expert workshop to examine and reflect on these barriers and facilitators for QI in EUs and identify which QI domains should be prioritised for development of QI guidance.

### Phase 1: qualitative interviews

#### Study setting

EUs in LMICs that were known to have attempted to implement or maintain QI initiatives. LMICs were defined as countries whose gross national income per capita was less than US$14 005, as per the World Bank country classification for 2024.^30^

#### Participants

Emergency physicians with experience in leading QI initiatives in EUs in LMICs and consenting to be interviewed were included as study participants. A purposive sampling strategy was employed to recruit participants with relevant expertise in QI implementation in these settings. Potential participants were identified in collaboration with the WHO technical team on ECO care, drawing on its global network to ensure appropriate experience and geographical diversity. 13 physicians were invited to participate. Sample size was guided by the principle of thematic saturation.^31^

#### Data collection

IDIs were conducted between October 2023 and February 2024. A topic guide (TG) was developed based on findings from previous studies^32 33^ and authors’ knowledge of delivery of QI and providing emergency care (online supplemental material 01). Questions in the TG focused on their most recent experiences as an emergency physician working in an EU and inquired about the QI practices in EUs and the barriers and facilitators encountered in implementing such activities. The TG was piloted prior to use. All IDIs were conducted virtually and recorded for post interview transcription. Interviews were conducted in English, given participants were proficient in the language. Data collection and analysis were undertaken concurrently such that the IDIs continued until thematic saturation was reached.^34^ Saturation was determined through an iterative approach of simultaneous data collection and analysis to identify new codes and themes; saturation occurred when no new codes or themes were identified in interviews.^31^

#### Data analysis

Audio recordings were transcribed and the transcripts were uploaded to NVivo software^35^ for coding and analysis. Interviews were analysed thematically following the approach described by Braun and Clarke^36^ and incorporating elements of the constant comparative method.^37^ Initially, a sample of interview transcripts was reviewed to generate inductive codes, focusing on identifying specific barriers and facilitators for QI initiatives in the LMIC EUs. This was followed by deductive coding applied based on the WHO Health Systems Framework.^38^ This enabled the identified barriers and facilitators coded to be aligned with the WHO Health systems framework, with further refinements made through discussion among authors to ensure consistency and accuracy. Findings were assessed quantitatively, considering the number of participants per code and qualitatively, using illustrative quotes to provide deeper insights. These identified barriers and facilitators were thereafter summarised and presented.

### Phase 2: workshop with technical experts

#### Workshop setting

Geneva, Switzerland.

#### Participants

Emergency physicians, other EU-related clinical specialists and emergency care nurses involved in EU care, recognised to be experts through clinical practice, research, leadership or policy in QI activities were invited to participate in the workshop. Participants representing different countries with diverse income levels and geographical regions were purposefully selected as suggested by the WHO technical team on ECO care. This approach was taken given our knowledge from the literature that QI efforts in the ED are scarce. Therefore, using WHO’s networks provided an efficient way of identifying participants who had delivered QI initiatives in the ED and could speak to the barriers and facilitators. The number of experts invited was capped at 26 due to logistical limitations.

#### Data collection and analysis

The 2-day workshop was structured around plenary presentations and discussions. Findings from the phase 1 qualitative study were presented, and participants were requested to reflect on and articulate their experiences of barriers and facilitators for QI in EUs. This was followed by a presentation and discussion on the WHO quality domains, after which questions were posed to the participants to understand their thinking about what measures could be used to assess each QI domain. The participants were allowed to respond with multiple responses, which they deemed were appropriate measures for assessing quality domains. The responses were captured as word-clouds through the voting app, Menti (Menti.com). The top three most common suggestions for measurement under each quality domain were identified and presented.

Finally, a presentation on QI processes in the EU and the current availability of WHO tools and guidance for QI (online supplemental material 02) were discussed. Thereafter, the participants were requested to rank the WHO quality domains based on their perceived feasibility to enhance the domains, using Slido (Slido.com). Participants were allowed three separate rounds of voting. In the first round, they voted for the domain they thought would be most feasible to enhance, while in the subsequent two rounds, they voted for the next most feasible domain to enhance, excluding their voted domains in the earlier rounds. At the end of the three rounds, the votes were compiled to identify the top three most feasible domains to change as perceived by the participants, and these were presented.

Outputs were additionally captured through meeting notes and transcripts of recorded meeting proceedings. Given that QI requires providers to identify, measure and change existing practices, and such change requires capability, opportunity and motivation, the study findings were synthesised and summarised by the research team focusing on:

1. Challenges of practicing QI in EU settings in LMICs.
2. Potential facilitators to overcome these challenges in EUs.
3. Perceived measures for assessing each of the WHO quality domains in the EUs.
4. Perceptions on the WHO QI domains that are most feasible to enhance in EUs.

The final report of the workshop was shared with all workshop participants for their comments and confirmation. No patient or public involvement was required for this study.

## Results

Eleven IDIs were completed before reaching thematic saturation in phase 1, and all 26 experts who were invited attended the expert workshop (table 1).

**Table 1:** Participant characteristics in the qualitative interviews study and the workshop

Current roles	Gender distribution	Represented countries
(A) Qualitative Study (phase 1)		
Specialist emergency physicians (N=8) Specialist in internal medicine and surgery in emergency units (N=1) Specialist emergency physicians currently working as technical advisors for the Ministries of Health (N=2)	Male - 8 Female - 3	Arab Republic of Egypt Federal Democratic Republic of Ethiopia Republic of India Republic of Liberia Islamic Republic of Pakistan Republic of the Philippines Republic of South Africa Republic of the Sudan Kingdom of Thailand Republic of Uganda Republic of Zambia

(B) Workshop of technical experts* (phase 2) [Table-fn T1_FN1]		
Specialist emergency physicians (N=19) Clinical specialists in internal medicine and surgery in emergency units (N=1) Critical care nurses (N=1) WHO technical experts in QI and ECO care (N=5)	Male - 11 Female - 15	Commonwealth of Australia Belize Arab Republic of Egypt Federal Republic of Germany Republic of Ghana Republic of India Republic of the Philippines Kingdom of Norway Republic of Rwanda Republic of South Africa Swiss Confederation Kingdom of Thailand Republic of Uganda United Kingdom of Great Britain and Northern Ireland United States of America

* Two specialist emergency physician participants were interviewees in the phase 1 qualitative study.

ECO, emergency, critical and operative; QI, quality improvement.

### Results of phase 1: qualitative interviews study

**Figure 1:**
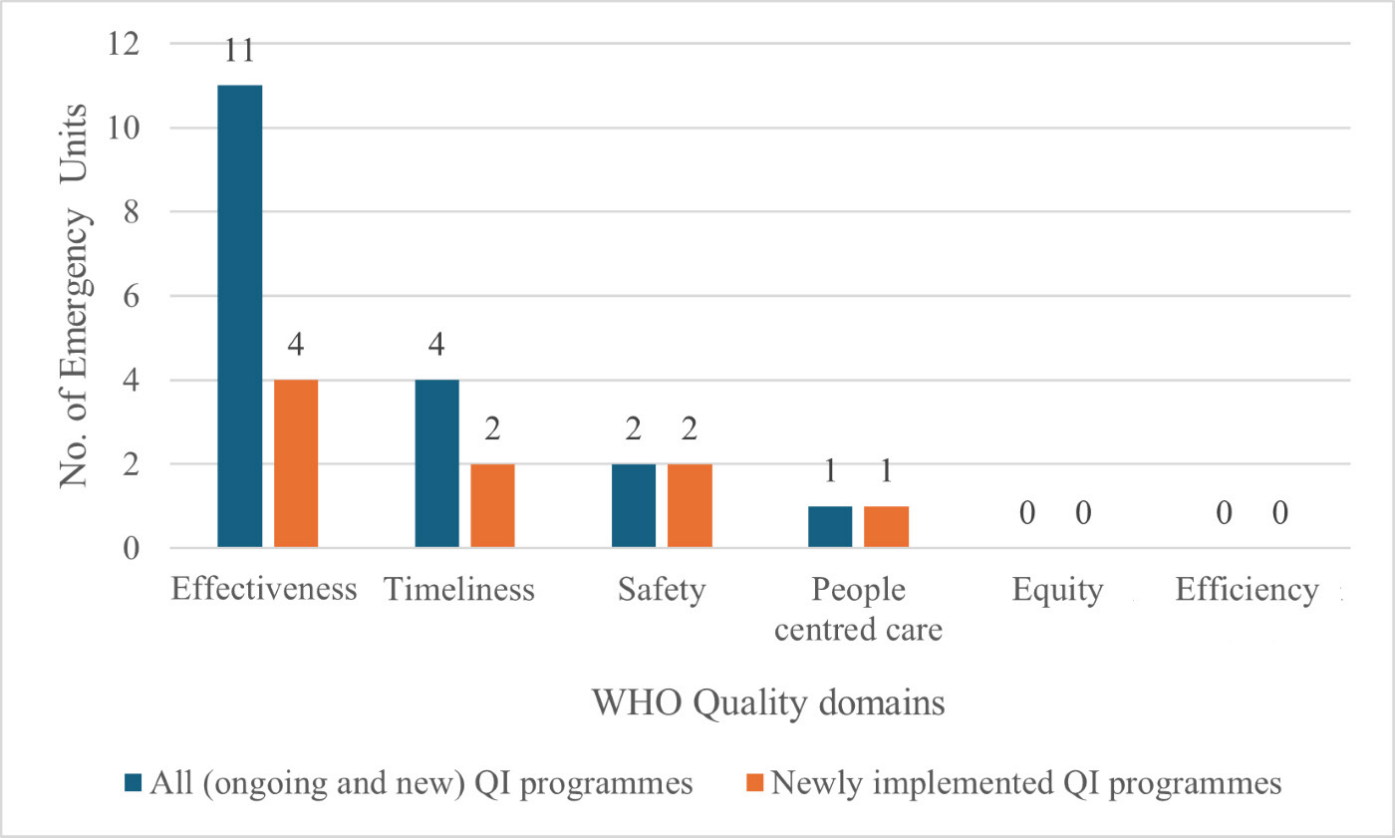
Number of EUs implementing QI initiatives as per WHO quality domains, and the number of EUs where new QI programmes were implemented by the participants (N=11). EU, emergency units; QI, quality improvement.

All 11 participants confirmed that QI activities were being conducted within their EUs. Among those activities, 25 specific longstanding initiatives were identified, primarily focused on improving effectiveness, timeliness, safety and person-centredness. Five participants reported new QI initiatives, initiated by them. All 11 EUs had QI initiatives related to effectiveness; none had initiatives focused on equity or efficiency (figure 1). Examples of initiatives conducted included reducing patient queues, initiating triage, staff training, obtaining patient feedback, conducting data-informed quality of care reviews and routine monitoring using key performance indicators (KPIs). Further details on QI initiatives are provided in online supplemental material 03.

#### Barriers to QI initiatives

Numerous barriers related to developing and implementing QI initiatives, categorised as per WHO Health systems building block framework^38^ are presented in table 2. The detailed findings, including relevant quotes specific to WHO QI domains are presented in online supplemental material 04—section A.

**Table 2:** Perceived barriers for quality improvement in EUs of LMICs

WHO building block	Common barriers perceived for implementing QI in EUs in LMICs
1. Service delivery	Lack of sustained availability of essential utilities, eg, 24 hours stable electricity supply or proper drainage systems.
	Lack of adequate and necessary infrastructure, eg, separate, secure space for EU.
	Lack of proper maintenance of infrastructure.
	Difficulties with coordinating and implementing QI initiatives into existing care delivery structures and processes, especially in relation to providing integrated care in EU and continuity of care of patients.
	Lack of feedback mechanisms to identify and strengthen the processes.
2. Health workforce	Inadequate numbers of staff due to poor recruitment.
	High dropout rates of clinical staff due to low salaries and incentives, lack of appreciation and poor career prospects.
	Lack of knowledge and skills related to QI and QI domains including patient safety, patient centredness, and timeliness of care.
	Poor prospects for QI-related capacity building.
	High workforce fatigue leading to resistance towards QI.
	Poor attitudes of staff towards patient care, safety and patient satisfaction.
3. Health information systems	Lack of connectivity, IT infrastructure, maintenance and trained support services.
	Challenges of data collection and analysis, especially due to existing paper-based manual data collection systems.
	Lack of patient safety monitoring systems for identifying safety issues.
	Difficulties in capturing patient issues for developing indicators to quantify and address issues effectively.
4. Access to essential medicines and equipment	Lack of availability of medical supplies and access to specialised investigations.
	Lack of necessary equipment for patient care such as blood pressure apparatus.
	Lack of maintenance of equipment making them unusable due to frequent breakdown.
5. Health financing	Government funding in public hospitals is barely adequate to cover basic recurrent expenses.
	Insufficient availability of funds for implementation and sustainability of QI initiatives.
	Redistribution of QI allocated funds for patient care activities.
6. Health leadership/governance	Lack of prioritising QI by hospital management, leading to administrative resistance and non-approval of financial support for QI initiatives.
	Perceptions prioritising the patient numbers cared for over the quality of care provided to patients.
	External influences affecting the activities of institutions, including the implementation of QI.
	Existence of poor QI culture and blame culture within the institutions.
	Lack of accountability framework and accreditation standards to promote QI.
	Lack of active involvement of departmental management in quality and safety committees.

EU, emergency unit; IT, information technology; LMICs, low- or middle-income countries; QI, quality improvement.

Of note, when discussing barriers, participants described challenges at different levels. Some explicitly referred to barriers that directly hindered QI delivery, while others highlighted broader challenges to delivering quality care which indirectly impeded ability to deliver QI. This highlights the complexity of separating challenges in delivering quality care from those specifically hindering QI implementation.

#### Facilitators for QI

Participants highlighted several factors which facilitated their attempts at implementing QI. These are summarised in table 3, with relevant quotes presented in online supplemental material 04—section B.

**Table 3:** Suggested facilitators for QI in EU

Main themes of facilitation	Activities
1. Use of guidelines and tools	The use of available, appropriate QI guidelines and tools, including QI related KPIs from the Ministry of Health, hospital administration or from external quality accreditation organisations.
	Assembling a local team (EU physicians and EU staff) to discuss and develop bespoke QI guidelines and indicators.
2. Improve communication	Enhancing communication between the EU team using simple mobile apps and texting services.
	Improving documentation and recording/filing of data to facilitate better data storage and utilisation.
3. Enhancing integrated care	Initiate and develop interunit guidelines on care of patients towards ensuring continuity.
4. Training and capacity development	Conducting formal and informal QI training for staff.
5. Optimising use of available resources	Rather than seeing a lack of essential medicines and equipment as a barrier to improving care quality, encouraging the use of alternative resources to improve timely delivery of patient care.
6. Attempting and being involved with initiating small scale QI initiatives	Planning and attempting small scale, minimal cost QI activities, which are easy to implement, monitor and sustainable, while causing minimal financial or human resource burden.
	Encouraging all staff and trainees to be involved with QI-related activities to create a culture of QI.
7. Promote external collaborations	Engaging in collaborations with other departments or external universities and funders to facilitate support for larger QI activities.

EU, emergency units; KPI, key performance indicator; QI, quality improvement.

### Results of phase 2: workshop with technical experts

#### Perceived measures for assessing quality domains in the EU

Summarised responses on measures to assess WHO QI domains are presented in table 4, with the word clouds and full results of responses presented in the online supplemental material 05. While some domains were clearly demarcated, the categorisations of others were blurred. For instance, timeliness of care was included as a measure to assess effectiveness of care and measures for efficiency were offered under measures of timeliness. Some participants presented domain definitions rather than methods for measurement.

**Table 4:** Measures suggested by workshop attendees for assessing the concepts of WHO quality domains

Quality domain	No of participants (No. of total responses)	Top three responses suggested for assessing QI (No. of specific responses)	WHO description of domain
Effectiveness	18 (38)	Clinical outcomes (11)	‘Providing evidence-based healthcare services to those who need them’
		Timeliness of care (5)	
		Adherence to clinical care guidelines (3)	
Patient safety	18 (44)	Safety practices (16)	‘Avoiding harm to people for whom the care is intended’
		Complication metrics (5)	
		Clinical outcome (4)	
Patient centredness	19 (44)	Patient preferences (10)	‘Providing care that responds to individual preferences, needs and values’
		Care quality metrics (9)	
		Patient satisfaction (7)	
Timeliness	18 (41)	Timeliness of care (24)	‘Reducing waiting times and sometimes harmful delays’
		Length of stay (5)	
		Quality of care (5)	
Equity	18 (43)	Accessibility (16)	‘Providing care that does not vary in quality on account of gender, ethnicity, geographic location, and socio-economic status’
		Non-discrimination (10)	
		Patient characteristics (5)	
Efficiency	19 (49)	Cost measures (15)	‘Maximizing the benefit of available resources and avoiding waste’
		Resources utilisation (12)	
		Competence (7)	
Integrated care	18 (49)	Process of integration (12)	‘Providing care that makes available the full range of health services throughout the life course’
		Communication (10)	
		Team effectiveness (7)	

QI, quality improvement.

#### Perceptions on feasibility of WHO quality domains to enhance

Safety (76.5%) was ranked by the workshop participants, as the most feasible domain to enhance, followed by effectiveness 52.9%. Patient centredness and timeliness of care were equally ranked (41.2%) as the third most feasible domain for enhancing. Efficiency (29.4%), integrated care (17.6%) and equitable care (11.8%) were ranked as less feasible to improve. All participants agreed that it would not be feasible to enhance all quality domains simultaneously.

#### Barriers and facilitators to implementing QI in the EU settings

Reflecting on findings from the qualitative interviews, workshop participants posed that barriers were primarily related to three key factors.

- First, EUs face a heavy patient load, making it difficult to implement QI initiatives effectively.
- Second, patient diagnoses are often unknown during their stay in the EU, adding complexity to clinical decision-making.
- Third, the EU is not the final point of care for patients but is instead deeply interconnected with other hospital services and external healthcare interactions.

Although not ranked in the top three barriers, many participants emphasised the importance of frontline healthcare staff safety in EUs. Given the high-pressure environment, ensuring that QI initiatives did not compromise the personal safety of healthcare providers was highlighted as a critical concern.

Suggested facilitators were generic to QI delivered in other units, but felt to be of particular importance in EU, given the aforementioned challenges. For example, ensuring QI programmes are developed with inputs from staff and patients was recognised as a way to enhance their local ownership and relevance. Participants highlighted the need for broad, adaptable improvement strategies rather than interventions reliant on specific clinical care pathways, allowing for quality improvements regardless of case mix and diagnoses. Working towards a minimum standard of care was suggested as an EU-specific facilitator. However, other participants preferred to work towards achieving any improvements from baseline. Incorporating assessments of quality of care delivered prior to patient arrival in the EU was considered important, but difficult to capture and change. Finally, participants agreed that ensuring QI initiatives are feasible in resource constrained EUs was essential for the sustainability of QI. Details are presented in online supplemental material 06.

## Discussion

This study explored the delivery and implementation of QI initiatives in EUs in LMICs, identifying barriers, facilitators and feasibility considerations for QI activities. Our findings provide insights into existing QI efforts and implementation challenges, emphasising the need for structured, adaptable tools and guidance to support and sustain QI efforts in EUs.

The interview data revealed that existing QI programmes primarily focused on effectiveness, safety and timeliness of care, aligning with findings of prior studies.^26–29^ However, patient-centredness was less frequently targeted, while equity and efficiency were absent, highlighting gaps in QI efforts. Effectiveness was the most frequently implemented QI initiative, yet barriers such as limited guidelines, lack of timely and accurate information and resource constraints significantly hindered the impact. Workshop discussions emphasised the need for substitute resources in LMIC settings to mitigate these challenges, particularly in the context of staffing shortages and the absence of structured QI capacity-building programmes.^39^

### Balancing the WHO quality domains in the EU

Workshop participants identified safety as the most feasible domain for improvement, reinforcing the global emphasis on patient safety in hospitals.^14 28 40^ They cited administrative, financial and reputational concerns as key drivers for prioritising patient safety, emphasising the need for targeted guidance to strengthen QI implementation in EUs. While safety remained a top priority, participants acknowledged that all QI domains are interdependent and essential for delivering high-quality care.^13^ Discussions highlighted the challenge of balancing these priorities, particularly in resource-constrained settings and underscored the need for a comprehensive approach to QI.

Patient-centredness in care was ranked third in feasibility for change, with several measurement approaches suggested. Patient-reported measures are considered reliable indicators,^41 42^ but workshop participants noted challenges in collecting and analysing patient issues and service reviews, indicating a need for EU-specific measures of patient-centredness. Timeliness of care was ranked equally feasible to improve by workshop participants; it was also the third most common focus of QI initiatives among interview participants’ hospitals. Numerous timeliness-based KPI were being used by interview participants as standardised measurement methods. The integrated nature of care in the EU, where services rely on other departments such as diagnostics and referral systems, emphasised the need for specific timeliness metrics which are possible to improve in EU settings. Equity and efficiency were largely absent from QI efforts in interviews with hospital providers despite equity’s recognised importance in universal health coverage.^43^ Considering the fact that emergency patients with time-critical health conditions are encountered at the EU, and in most instances, they require fast-paced, quick thinking and decision-making processes, the key focus is to prevent mortality and overcome the critical situation.^44^ In such high-pressure encounters, the need for saving a life becomes more of a priority than considering inequity in care based on discriminatory factors. Hence, the possibility of lower consideration for equity in care among our participants. Indeed, in our study of injured patients admitted to hospitals across four LMICs, we found that patient clinical outcomes as well as measures of patient centredness were remarkably equitable.^45^

### Need for clearer definitions and guidance on WHO quality domains

Menti results on suggested measures for each WHO quality domain show that there is some confusion among participants about the definition of each domain, eg, efficiency was often misinterpreted as timeliness, and competence was suggested as a measure of efficiency. However, this confusion also reflects the overlapping and interdependent nature of WHO quality domains, particularly timeliness, effectiveness and safety. Workshop participants faced challenges towards defining measurable indicators for integrated care. These findings mirror those from other healthcare settings where these concepts were often thought to be interconnected and difficult to distinguish clearly.^27^ While none of our workshop participants thought that all quality domains were equally important, the interdependency of the domains means that improvement in quality in any domain may have beneficial effects on others.^46^ Lack of clarity around the quality domains suggests that future guidance should emphasise understanding all quality domains, ensuring that QI efforts do not neglect any. There should also be suggestions for measurements appropriate for each and all domains, and guidance should illustrate completing the QI loop, for multiple domains, broader than the usual ones of effectiveness and safety.^47^

### Quality enhancement through WHO Health systems framework

Our findings indicate that barriers to QI in EUs align with WHO’s Health systems framework,^38^ spanning infrastructure deficits, workforce limitations, governance weaknesses and system-wide inefficiencies. Context-specific challenges in LMICs, such as unreliable electricity, inadequate infrastructure and equipment shortages, underscore the need for QI tools that accommodate resource constraints while maintaining measurable improvements in care quality. Additionally, workforce shortages and a lack of QI training highlight the importance of developing accessible training materials, toolkits and implementation frameworks to equip frontline healthcare workers with the skills needed to integrate QI into routine practice. While some of our findings align with prior studies,^3 15 48^ there is limited research exploring all aspects of QI implementation across all WHO quality domains.

Despite these barriers, several facilitators were identified that could enhance QI programme development and implementation. The use of QI guidelines and locally developed protocols, tailored to EU-specific workflows, was considered critical. This suggests that new guidance should build on existing frameworks while allowing for local customisation, ensuring that QI tools remain practical, adaptable and relevant to EU settings. Strengthening internal communication within EUs and across hospital departments was also highlighted by participants as an important strategy for integrating QI into broader healthcare systems. In resource-constrained settings, small-scale, resource-efficient QI initiatives were seen as effective starting points, allowing gradual implementation without overwhelming existing systems, thereby minimising resistance from the overburdened EU staff and the hospital administration. Seeking external collaborations with universities, non-governmental organisations and international organisations to secure funding and technical support could be effective for implementing and sustaining broader QI interventions.

### Limitations

Our study has several limitations. We selected participants through WHO’s networks. This could have biased the selection to those participants with more success in delivering QI in the ED. However, given that our knowledge of the literature suggests that QI initiatives in the EU are scarce, involving WHO was a pragmatic approach to select participants who we knew had attempted QI and could speak to barriers and facilitators experienced. As a result of our selection process, most participants were senior emergency clinicians, which may have introduced bias in emphasising certain QI domains over others, potentially shaping the prioritisation of effectiveness while under-representing perspectives on other aspects of QI. Though every attempt was made to include study participants to ensure diversity in geography, sex and experience, finding eligible participants was very much challenging. The IDIs primarily included participants from Africa and Asia, with no representation from the Pacific and America regions, potentially limiting the generalisability of findings to other LMIC settings. However, this limitation was partially mitigated by the broader geographical representation of participants in the expert workshop. While efforts were made to capture a range of perspectives, differences in health system structures, policy environments and available resources may have influenced participants’ experiences with QI. Finally, because data were collected through semi-structured interviews and a workshop, findings are based on self-reported experiences and perceptions. Our participants were QI experts, and our data may not have captured the concerns of those less engaged in QI efforts in LMICs.

### Recommendations

This study highlights the need for LMIC-specific guidance and tools to support QI implementation in EUs. The QI resources must address existing gaps, particularly in equity, efficiency, patient-centredness and integrated care, while providing suggestions to overcome infrastructure, workforce and governance-related challenges. The development of adaptable QI tools—including training programmes and standardised implementation guides—will be essential in ensuring sustained improvements in EU care quality across diverse LMIC settings. Further research on understanding of quality improvement perspectives of less-engaged staff including health administrators on enhancing care quality in EUs and exploring how a culture of providing high quality healthcare within the EUs could be created through sensitisation of EU staff would provide further evidence for developing effective QI programmes. Developing frameworks for capturing and analysing patient suggestions and problems especially within EUs would be highly beneficial towards strengthening the care quality in EUs.

## Conclusion

EUs are a unique environment within health facilities, which encounter time-critical patients who require urgent, but effective and safe interventions for survival, while ensuring patient centred, efficient and equitable care. However, QI appears to focus mainly towards enhancing the effectiveness and timeliness of care in EUs in LMICs. Despite the numerous barriers originating from both internal and external factors, opportunities exist, supported by internal and external facilitators, to enhance care quality across all seven quality domains. Process enhancing activities requiring minimal resources and implementation time can have a meaningful impact as their cumulative effects lead to major improvements in care quality. However, practical measures and indicators are essential to monitor progress effectively and ensure that all QI domains are addressed. Such tools will enable a more systematic and balanced approach to QI, fostering sustainable improvements in emergency care delivery in low resourced settings.

## Supplementary material

10.1136/bmjoq-2026-004186 online supplemental file 1

## Data Availability

Data are available upon reasonable request.
